# Genomic and phenotypic characterization of finger millet indicates a complex diversification history

**DOI:** 10.1002/tpg2.20392

**Published:** 2023-11-20

**Authors:** Jon Bančič, Damaris A. Odeny, Henry F. Ojulong, Samuel M. Josiah, Jaap Buntjer, R. Chris Gaynor, Stephen P. Hoad, Gregor Gorjanc, Ian K. Dawson

**Affiliations:** ^1^ The Roslin Institute and Royal (Dick) School of Veterinary Studies University of Edinburgh, Easter Bush Research Centre Midlothian UK; ^2^ Scotland's Rural College (SRUC) Kings Buildings Edinburgh UK; ^3^ International Crops Research Institute for the Semi‐Arid Tropics ICRAF House Gigiri Nairobi Kenya; ^4^ Department of Horticulture University of Georgia Athens Georgia USA

## Abstract

Advances in sequencing technologies mean that insights into crop diversification can now be explored in crops beyond major staples. We use a genome assembly of finger millet, an allotetraploid orphan crop, to analyze DArTseq single nucleotide polymorphisms (SNPs) at the whole and sub‐genome level. A set of 8778 SNPs and 13 agronomic traits was used to characterize a diverse panel of 423 landraces from Africa and Asia. Through principal component analysis (PCA) and discriminant analysis of principal components, four distinct groups of accessions were identified that coincided with the primary geographic regions of finger millet cultivation. Notably, East Africa, presumed to be the crop's origin, exhibited the lowest genetic diversity. The PCA of phenotypic data also revealed geographic differentiation, albeit with differing relationships among geographic areas than indicated with genomic data. Further exploration of the sub‐genomes A and B using neighbor‐joining trees revealed distinct features that provide supporting evidence for the complex evolutionary history of finger millet. Although genome‐wide association study found only a limited number of significant marker‐trait associations, a clustering approach based on the distribution of marker effects obtained from a ridge regression genomic model was employed to investigate trait complexity. This analysis uncovered two distinct clusters. Overall, the findings suggest that finger millet has undergone complex and context‐specific diversification, indicative of a lengthy domestication history. These analyses provide insights for the future development of finger millet.

## Core Ideas

- 8778 SNPs and 13 agronomic traits characterized a panel of 423 finger millet landraces.
- Four clusters of accessions coincided with major geographic areas of finger millet cultivation.
- A comparison of phenotypic and genomic data indicated a complex diversification history.
- This was reflected in an analysis of allotetraploid finger millet's separate sub‐genomes.
- New knowledge for intra‐ and inter‐regional breeding is provided.

**Abbreviations**
AMOVA: analysis of molecular variance
CV: coefficient of variation
DAPC: discriminant analysis of principal components
DArTseq: Diversity Arrays Technology sequencing
GRM: genomic relationship matrix
GWAS: genome‐wide association study
LD: linkage disequilibrium
MAF: minor allele frequency
MLM: mixed linear model
MTA: marker‐trait association
NJ: neighbor‐joining
PCA: principal component analysis
RR‐BLUP: ridge regression‐best linear unbiased genomic prediction
SNP: single nucleotide polymorphism

## 1 INTRODUCTION

Addressing global concerns surrounding human and environmental health, including malnutrition and unsustainable monoculture production systems, necessitates the crucial objective of diversifying crop production (Bančič et al., 2021; von Grebmer et al., 2014). Achieving this objective requires a renewed emphasis on under‐researched crops, commonly referred to as orphan crops. Some of these orphan crops are rich in micro‐ and macro‐nutrients and can complement other crops in food production systems, even when farming conditions are adverse (Kamenya et al., 2021; Mustafa et al., 2019). Beyond their inherent value, orphan crops boast complex demographic histories that can illuminate broader aspects of crop domestication and diversification (Meyer & Purugganan, 2013). Helping to overcome traditional challenges stemming from the high cost of generating genomic resources, cost reductions in high‐throughput sequencing during the past couple of decades have opened up new avenues for genetically analyzing orphan crops (Jamnadass et al., 2020).

**FIGURE 1:**
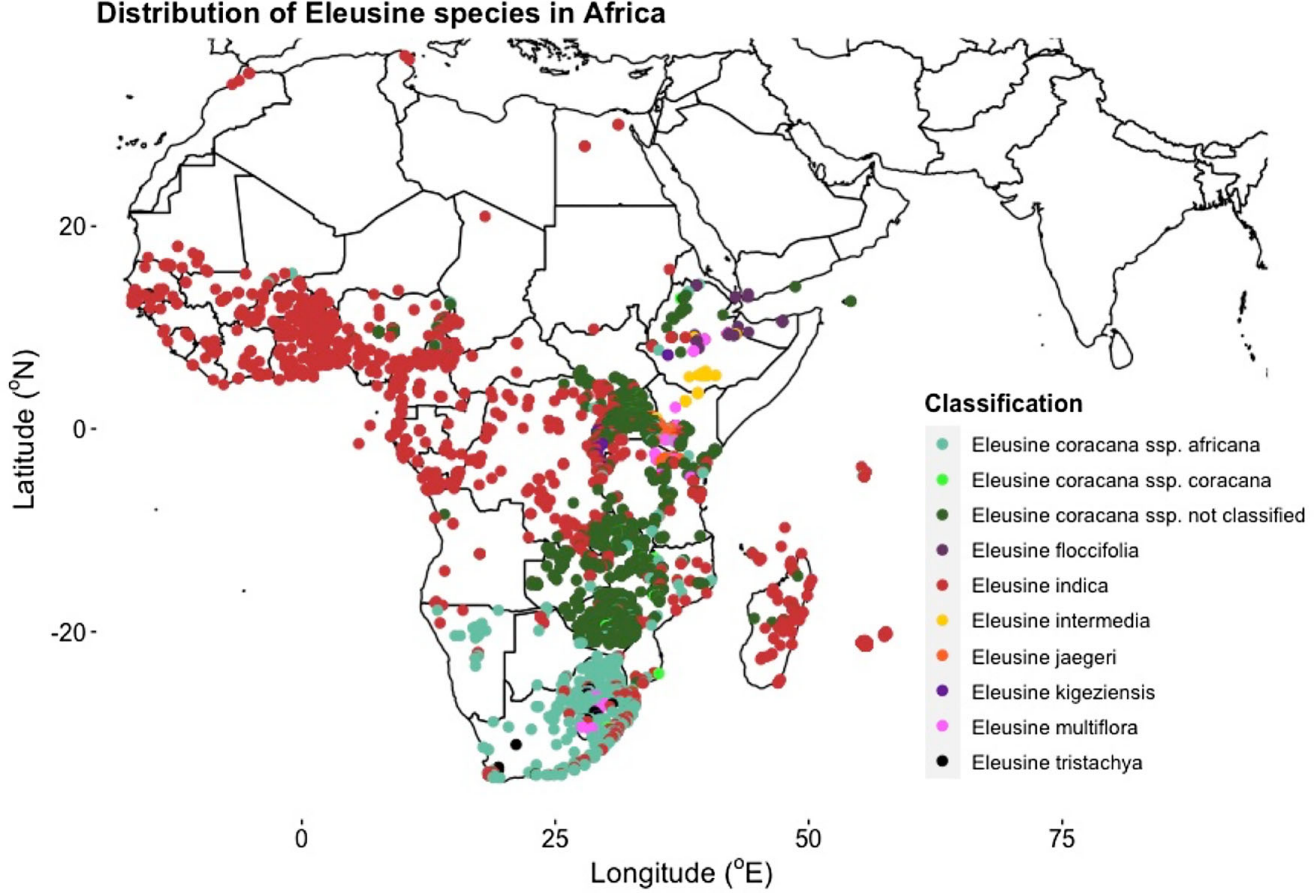
Distribution of eight *Eleusine* species across the African continent. A total of 9250 known locations of *Eleusine* were extracted from the Global Biodiversity Information Facility (GBIF; https://www.gbif.org/). Some *Eleusine coracana* entries in GBIF were not classified at the subspecies level (“not classified” in key).

Finger millet (*Eleusine coracana* (L.) Gaertn. subsp. *coracana*; Poaceae, subfamily Chloridoideae) is an annual small‐grained cereal and an orphan crop with an important role in smallholder food production systems in parts of Africa and Asia. The crop exhibits a range of appealing characteristics, including a rich nutritional profile, versatility in food usage, good storage properties, high market value, adaptability to poor production conditions, and flexibility in integrating into various farming approaches (Odeny et al., 2020; Sood et al., 2019). According to de Wet et al. (1984), the domestication of finger millet, an allotetraploid crop with a reported disomic inheritance (2*n* = 4x = 36; genome constitution AABB), occurred approximately five millennia ago in either Uganda or the Ethiopian highlands of East Africa, with its progenitor being the wild *E. coracana* subsp. *africana*. Subsequently, the domesticated subsp. *coracana* spread to southern Africa while broadly maintaining sympatry with the subsp. *africana* wild form, and while remaining in proximity to other *Eleusine* species (see a distribution of the eight *Eleusine* species in Africa depicted in Figure 1). de Wet et al. (1984) consider that the crop was introduced to India around three millennia ago, from where it was then dispersed further, including to Nepal. As a result, East Africa is considered the primary center of diversity, with India an important secondary center. The immediate wild progenitor of finger millet is believed to have emerged in East Africa by hybridization between two diploid species: *E. indica*, which still widely exists across Africa and is recognized as the maternal donor of finger millet's A sub‐genome; and a second as yet still unknown and potentially extinct B sub‐genome donor (Liu et al., 2014; Zhang et al., 2019).

**Core Ideas**

- 8778 SNPs and 13 agronomic traits characterized a panel of 423 finger millet landraces.
- Four clusters of accessions coincided with major geographic areas of finger millet cultivation.
- A comparison of phenotypic and genomic data indicated a complex diversification history.
- This was reflected in an analysis of allotetraploid finger millet's separate sub‐genomes.
- New knowledge for intra‐ and inter‐regional breeding is provided.

In Africa and Asia, finger millet is primarily cultivated by subsistence farmers who rely on landrace varieties, and the genetic improvement of the crop has been limited, highlighting its status as an orphan crop. The complex biology of finger millet, along with its predominantly self‐pollinating nature and small flowers, poses challenges for artificial crosses and breeding efforts (Dida & Devos, 2006). Additionally, the genomic characteristics of finger millet as an allotetraploid, with an unknown B sub‐genome donor, have hindered the development of genomic resources. The difficulty in accurately distinguishing between sub‐genomes, and consequently the potential for inaccurate identification of homologous versus homeologous single nucleotide polymorphism (SNP) positions, has been a concern (Hatakeyama et al., 2018; Hittalmani et al., 2017). Landrace varieties, however, provide important opportunities to explore crop domestication. Many have been sampled for conservation in genebanks (Upadhyaya et al., 2006), and these accessions are available for use in breeding programs. These landrace accessions serve as a resource to investigate the crop's diversification history, an area of research that has received limited attention thus far.

This paper uses a combination of genomic and phenotypic data to explore a broad panel of 423 finger millet landrace accessions sampled across its main cultivation regions in Africa and Asia. We make use of Diversity Arrays Technology sequencing (DArTseq) (Sansaloni et al., 2011) SNP markers positioned onto finger millet's sub‐genomes using a recent genome assembly (https://phytozome‐next.jgi.doe.gov/info/Ecoracana_v1_1). The objective of our study is to characterize the crop's genomic and phenotypic variation to explore the diversification process and to provide insights for future breeding across its main cultivation range. We here investigate multiple features of finger millet variation, including the geographic structuring of genomic and phenotypic diversity, sub‐genome‐specific diversity profiles, germplasm migration events among geographic areas, and genetic architecture and selection patterns for agronomic traits. This analysis provides insights for the future development of the finger millet crop.

## 2 MATERIALS AND METHODS

### 2.1 Plant material

This study presents a comprehensive dataset of genotyped and phenotyped finger millet landrace accessions, with accessions originating from the crop's primary cultivation regions in Africa and South Asia. The accession panel initially contained 458 entries (later reduced to 423 entries for analysis) that as well as encompassing the main cultivation regions included a small number of accessions collected more widely (Table S1). Out of the total, 19 accessions were designated as “other,” either collected from outside the main cultivation regions or possessing unknown origins. The accessions in the panel were sourced from the ICRISAT genebank's core collection of finger millet, assembled by Upadhyaya et al. (2006), and have been used to introduce novel traits into ICRISAT's African finger millet breeding program. Most of the core collection was initially sampled directly from farmers’ fields, although sometimes accessions were sampled from local markets (see, e.g., Rao, 1980).

### 2.2 Collecting and processing genomic data

Leaf tissue was taken from single individuals of each of the 458 accessions, grown in a greenhouse at ICRISAT in Nairobi and dried with silica gel. Genomic DNA (gDNA) was extracted from finely ground leaf using the ISOLATE II Genomic DNA Kit (Bioline Pty Ltd) and according to the manufacturer's instructions. The purity and quantity of extracted gDNA was determined by gel electrophoresis and a Qubit 2.0 Fluorometer (Life Technologies, Carlsbad, CA), with a final dilution of gDNA to 50 ng/μL. Genomic DNA was processed at the Integrated Genotyping Service and Support (IGSS) facility at the Bioscience eastern and central Africa–International Livestock Research Institute (BecA–ILRI) hub in Nairobi. The methods for library construction, DArTseq, data generation, and initial output quality control steps were described previously by Sansaloni et al., 2011. The SNP positions were assigned using the v1.1 finger millet genome assembly (https://phytozome‐next.jgi.doe.gov/info/Ecoracana_v1_1), which was recently detailed in the study of Devos et al. (2023).

The IGSS facility generated 70,906 raw SNPs, which were then quality filtered using TASSEL (version 5.0; Bradbury et al., 2007) as illustrated in Figure S1. The accessions and SNPs were filtered using a minimum call rate of 70%, which reduced the initial sample size of 458 accessions to 423 (Table S1). This constituted the final accession panel for later data analyses. Further, only SNPs with a minor allele frequency (MAF) >0.01 were retained. SNPs with a heterozygosity level greater than 2pq×(1−F) were also removed, where *p* and *q* are the frequencies of the two allele states, and F is the inbreeding coefficient, for which a value of 0.5 was chosen due to the self‐pollinating nature of finger millet. This filter was applied to remove SNP calls that most likely originated from incorrectly collapsing homeologous positions across the two sub‐genomes.

### 2.3 Measuring linkage disequilibrium decay along chromosomes

Linkage disequilibrium (LD) decay (r^2^) between all pairwise intra‐chromosomal SNP combinations was calculated using a full correlation matrix either uncorrected or corrected for bias, based on underlying genetic structure across the accessions. Calculations of *r* ^2^ were performed in the R package *LDcorSV* (Mangin et al., 2012). Pairwise *r* ^2^ values were then plotted against chromosomal physical distance. The decay curve was fitted based on Hill and Weir (1988) using R code from Marroni et al. (2011) and was then used to estimate the distance at which *r* ^2^ decreased to 0.2. LD decay was estimated for each chromosome separately, for each sub‐genome and for the entire genome.

### 2.4 Genetic structure, differentiation, and diversity

#### 2.4.1 Genome‐wide genetic structure

Four approaches were used to characterize and visualize genetic structure of the panel. First, principal component analysis (PCA) and discriminant analysis of principal components (DAPC) were undertaken using R packages *ade4* (Dray & Dufour, 2007) and *adegenet* (Jombart, 2008; the *find.clusters* function). The optimum principal component (PC) and cluster (*k*) numbers for PCA were set based on the change in the curve shape of profiles (Figures S2 and S3). Second, a genomic relationship matrix (GRM) was constructed using a centered‐identity‐by‐state method (Endelman & Jannink, 2012) in TASSEL. A heatmap of the GRM, calculated using the unweighted pair group method with arithmetic mean (UPGMA) cluster algorithm, was visualized using the *heatmap* R function (R Core Team, 2019). Third, an unweighted neighbor‐joining (NJ) tree (Saitou & Nei, 1987) was constructed using TASSEL and plotted with R package *phangorn* (Schliep, 2011). NJ analysis was applied to both SNPs across the entire genome and pooled at the sub‐genome level. Fourth, accessions were assigned to their genetic clusters using R packages *rworldmap* (South, 2011) and *ggplot2* (Wickham, 2009) to visualize the country‐level geographic distribution.

#### 2.4.2 Detection of introgression and gene flow

Treemix (Pickrell & Pritchard, 2012) analysis was performed to infer likely evolutionary history and identify possible introgression events among groups of accessions. Accessions were assigned to four geographic areas of sampling that we term “East Africa,” “Southern Africa,” “India,” and “Nepal” (Table S1). For simplification, the small number of accessions sampled from countries outside these locations (9 of all 404 “known”‐location accessions) were assigned to the most proximate of the defined four areas. The 19 accessions designated as “other” (see Section 2.1) were excluded from geographic area analysis. Table S1 provides complete information, but, in brief: the area “East Africa” included a small number of additional accessions (*N* = 5) from Nigeria and Senegal; and “India” included a few extra lines (*N* = 4) from Sri Lanka, the Maldives, and Pakistan. Using Treemix, maximum‐likelihood population trees were constructed based on genome‐wide SNPs using blocks of 50 SNPs and “East Africa” rooted as an out‐group. The number of tested migration events was varied from zero to three. Bootstrap replicates were generated using 50 SNPs to evaluate the robustness of tree topology, and Treemix R plotting functions were used to visualize results.

#### 2.4.3 Chromosome‐level nucleotide diversity and differentiation

Genetic diversity and differentiation were analyzed at the chromosome level for both sub‐genomes according to the four geographic areas (areas as explained above). For each area, π (Nei & Li, 1979) was calculated as the estimator of diversity and pairwise *F*_ST_ values (Weir & Cockerham, 1984) as the estimator of differentiation, using VCFtools (Danecek et al., 2011) and a 1‐Mb non‐overlapping sliding window. Genetic differentiation estimates were calculated for all six possible pairwise combinations of geographic areas. Results were plotted against chromosomal positions using R package *ggplot2*.

#### 2.4.4 Summarized gene diversity and differentiation statistics

Genome‐wide gene diversity and differentiation were summarized for the four geographic areas. Genome‐wide gene diversity (H; Nei, 1973) and pairwise *F*_ST_ values based on all high‐quality SNPs were calculated with R package *hierfstat* (Goudet, 2005). The H values were computed for the four defined genetic clusters (see above) of finger millet accessions (these approximate the four defined geographic areas, as will be explained below). The analysis of geographic areas was further extended to consider genome‐wide markers and diversity at the sub‐genome level. Last, an analysis of molecular variance (AMOVA) that partitioned genetic variation within and among the geographic areas (or clusters), based on 100,000 permutations, was performed using R package *pegas* (Paradis, 2010).

#### 2.4.5 Collecting and analyzing phenotypic data

To assess phenotypic variation in finger millet, the panel of 458 accessions was evaluated for 13 agronomic traits (Table S2). Accessions were grown in a single trial using a randomized complete block design with two replications. The trial was conducted over the long rainy season of 2015 at the Kenya Agricultural and Livestock Research Organization field station at Kiboko in Eastern Kenya (coordinates: 2° 20′ N, 37° 45′ E; altitude: 960 m; annual temperatures: min. 16.6°C, max. 29.4°C, average 23.0°; sandy clay loam calcareous soil). Seeds of each accession were drilled in 2‐m long plots of two rows spaced 50 cm apart. Within rows, plants were thinned after establishment to a spacing of 10 cm. Five plants from each plot were randomly selected for recording trait phenotypes, and plot‐level data were collected for yield‐related traits (Table S2). The traits measured included features of morphology, physiology, and yield that are important for crop production and the integration of finger millet in mixed crop farming systems (Dawson et al., 2019), including in intercrop systems (Brooker et al., 2015), that are of particular interest in breeding research (Bančič et al., 2021).

Phenotypic data, initially screened for outliers and errors, was used to obtain variance parameters and best linear unbiased estimates (BLUEs) with the following linear mixed model:

$$y_{\mathrm{ikn}}=\;\mu +r_{k}+g_{i}+b_{\mathrm{kn}}+e_{\mathrm{ikn}},$$

where $y_{\mathrm{ikn}}$ is the vector phenotypes of the *i*th accession tested in the *k*th replication (*k* = 1, 2) and in the *n*th block (*n* = 1,…, *n*); μ is the overall mean; $r_{k}\;$is the fixed effect of replication; $g_{i}$ is the random genotype effect with $g_{i}∼\;N\left(0,\sigma_{g}^{2}\right)$; $b_{\mathrm{kn}}$ is the random block effect with $b_{\mathrm{kn}}∼\;N\left(0,\sigma_{b}^{2}\right)$; and $e_{\mathrm{ikn}}$ is the random residual with $e_{\mathrm{ikn}}∼\;N\left(0,\sigma_{e}^{2}\right)$. $\sigma_{g}^{2}$, $\sigma_{b}^{2},$ and $\sigma_{e}^{2}$ are variances associated with accessions, blocks, and residuals. To obtain the BLUEs for each phenotypic trait, accessions were treated as fixed effects, employing the same model. The models were fitted using the R package *ASReml‐R* (version 4.1.0.90; Butler et al., 2017). Broad‐sense heritabilities were estimated as $H^{2}=\frac{\sigma_{g}^{2}}{\sigma_{g}^{2}+\frac{\sigma_{e}^{2}}{r}}$ for each trait. Distributions of BLUEs for each phenotypic trait and Pearson's correlation coefficients (r; Pearson, 1895) between all pairwise combinations were calculated and visualized using R (R Core Team, 2019). The coefficient of variation (CV) for each trait was calculated as $(\sigma /\bar{x})\times 100\%$ using BLUEs.

PCA was also applied to the BLUEs to explore the underlying phenotypic structure using the R package *factoextra* (Kassambara & Mundt, 2020). Variation of BLUEs within the defined geographic areas and genetic clusters was also visualized using boxplots.

### 2.5 Trait genetic architecture and further genomic–phenotypic comparison

#### 2.5.1 Genome‐wide association analysis

To test each SNP's effect on the 13 phenotypic traits, a genome‐wide association study (GWAS) was performed in TASSEL using a linear mixed model that corrected for genetic structure and cryptic relatedness (Yu et al., 2006). BLUEs calculated for each trait were taken as phenotype, and 8778 SNPs were taken as genotype. The model estimated variance components only once using the P3D method. A Bonferroni threshold of 5% was applied to account for multiple testing to declare significant marker‐trait associations (MTAs). Manhattan and quantile–quantile (Q‐Q) plots of the GWAS results were visualized with R package *qqman* (Turner, 2014). Putative candidate genes were selected using a 25‐kb genomic interval both upstream and downstream of a significant MTA. The interval was queried against the current finger millet genome assembly using the Integrated Genomics Viewer (IGV, v. 2.82; Robinson et al., 2011).

#### 2.5.2 Clustering SNP effects

To further evaluate the genetic architecture of the 13 phenotypic traits, dendrogram ordering was applied to the distribution of SNP effects derived from a ridge regression model (RR‐BLUP) across traits (Kooke et al., 2016). BLUEs of each trait were scaled by their standard deviation and taken as phenotypes, and the genotype matrix consisted of 8778 imputed SNPs. A *k*‐nearest neighbor imputation of the SNP matrix was performed in TASSEL using default parameters, and RR‐BLUP models for each trait were fitted with R package *AlphaMME* (https://github.com/gaynorr/AlphaMME). SNP effects from the RR‐BLUP model were then used to: first, calculate pairwise Euclidean distances over the first five mathematical moments in R package *moments* (v0.14; Komsta & Novomestky, 2015); and, second, construct a dendrogram from the Euclidean distance matrix using the UPGMA cluster algorithm with the *hclust* R function (R Core Team, 2019).

#### 2.5.3 Comparison of phenotypic and genome‐wide gene diversity by geographic area

A simple yet useful way to shed light on the particular selection histories of crops as they take different diversification pathways within specific geographic contexts is to compare phenotypic diversity levels with levels of underlying genome‐wide gene diversity. Here, a straightforward approach was adopted for initial comparisons that involve individual phenotypic trait CV values and gene diversity (H) values for genome‐wide SNP data. A ratio CV/H was calculated as the comparator for each of the four defined geographic areas to check primarily for rank differences across areas that may indicate particular phenotypic selection pressures by area. This type of approach is exemplified classically in studies of the diversification of tomato, where high phenotypic variation observed at specific traits is accompanied by overall underlying genomic diversity bottlenecks (Rodríguez et al., 2011).

## 3 RESULTS

### 3.1 Genome‐wide SNP data cover the entire finger millet chromosome complement but occur at a higher density in the A sub‐genome

The IGSS facility generated 70,906 raw SNPs, which were filtered into a final marker set of 8778 SNPs for 423 accessions for subsequent analyses. Among these, 8096 SNPs out of the total of 8778 had been previously mapped to chromosomes (Table S3). These SNPs spanned the entire genome but SNP density was higher on the A sub‐genome chromosomes (Table 1; approx. twice the density of markers of the B sub‐genome). The mean SNP density across both sub‐genomes was ∼8.6 per Mb. The A sub‐genome chromosomes are also consistently shorter (Figure S4). Consistent with expectations of significantly lower recombination in central chromosomic regions of selfing cereals (e.g., Bustos‐Korts et al., 2019), SNP density was generally considerably higher toward the ends of chromosomes (Figure S4). The proportion of markers removed from the initial raw data due to likely being homeologous was a relatively high 3.9% (2733 out of 70,906; markers above the red curve in Figure S1e). Therefore, precautions with homeologous markers are indicated for finger millet. The observed heterozygosity for the markers in the final set of 8778 SNPs was very low (median 0.048 and mean 0.057; Table S3), indicating that most finger millet accessions are highly inbred.

**TABLE 1:** Distribution of single nucleotide polymorphisms (SNPs) by finger millet chromosomes. SNP density per chromosome was calculated for individual 1 Mb non‐overlapping windows.

**Chromosome**	**Number of SNPs**	**Chromosome length (Mb)**	**SNP density (per Mb)**
1A	742	57.13	12.4
2A	683	60.90	11.1
3A	570	53.87	10.9
4A	618	39.68	15.4
5A	1057	66.30	15.4
6A	357	59.47	5.9
7A	553	48.45	11.2
8A	307	47.22	7.6
9A	486	45.36	11.6
**Sub‐genome A**	5373	53.15	11.3
1B	196	72.49	3.7
2B	323	70.83	5.3
3B	136	63.91	2.9
4B	158	50.88	2.9
5B	659	80.22	7.8
6B	244	74.22	5.0
7B	267	58.85	7.1
8B	294	60.62	5.4
9B	446	62.05	7.4
**Sub‐genome B**	2723	65.20	5.7
**Scaffolds**	682		
**Whole genome**	8778	1072.44	8.6

### 3.2 Linkage disequilibrium decay along chromosomes shows expected patterns for both sub‐genomes

The calculations indicate the expected overall pattern of LD decay and the importance of correcting for underlying genetic structure among accessions. For the genome as a whole, *r* ^2^ decayed to 0.2 at a distance of 106 kb when correcting for genetic structure compared to a distance of 1.77 Mb for the naïve model. Decay was slower for the B sub‐genome (*r^2^* = 0.2 at 168 kb, with correction) than the A sub‐genome (*r^2^* = 0.2 at 88 kb) and varied markedly across chromosomes within sub‐genomes (Figure S5). Overall, the relatively slow rate of LD decay was consistent with other self‐pollinating crops (Flint‐Garcia et al., 2003), including other millets (Jaiswal et al., 2019).

### 3.3 Genome‐wide genetic structure reveals strong geographic differentiation

**FIGURE 2:**
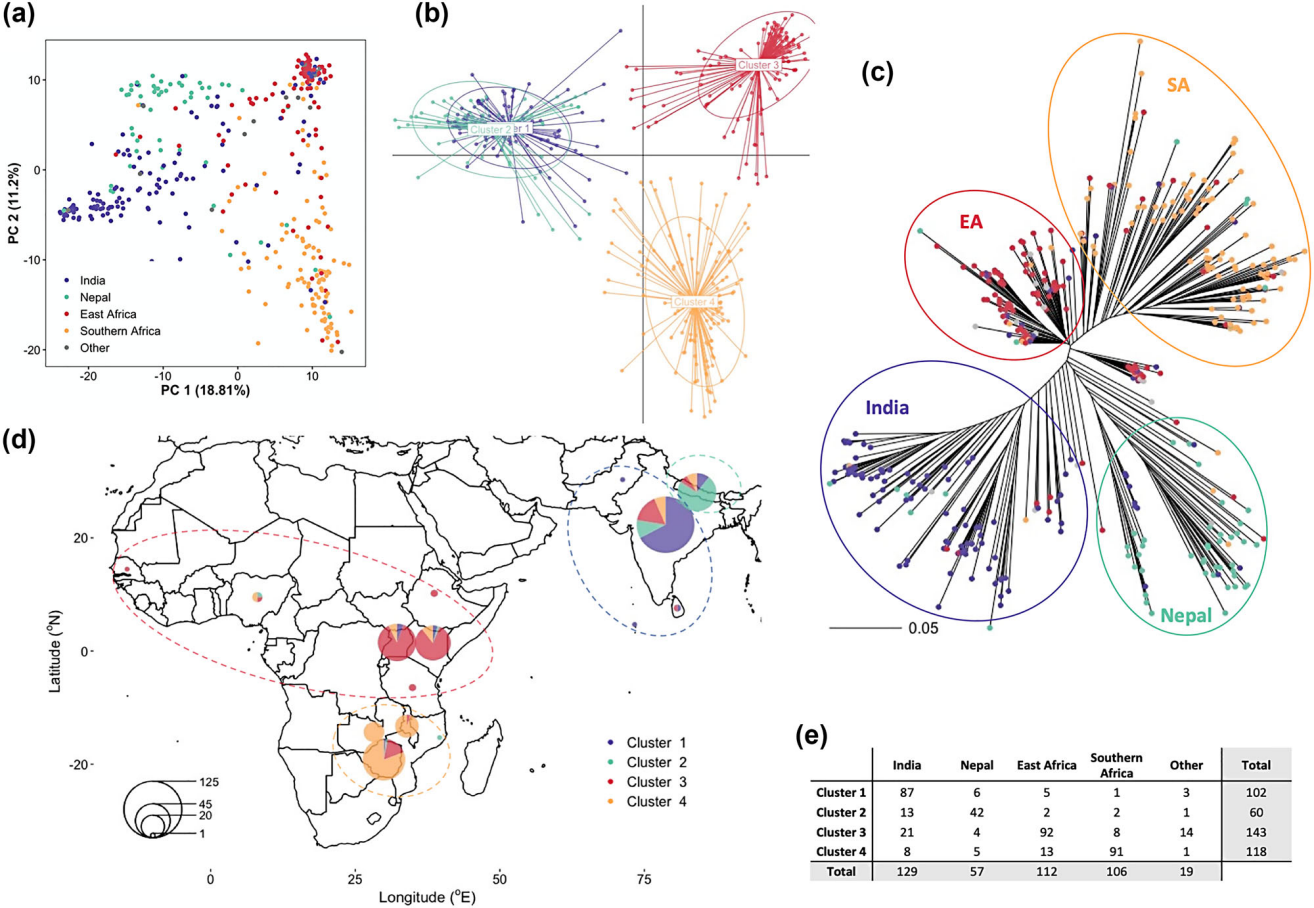
Geographical distribution and genetic structure of 423 finger millet accessions. (a) Score plot using the first two principal components (PCs) illustrates the genetic structure of finger millet colored according to geographic areas. (b) Genetic clusters identified by discriminant analysis of principal components (DAPC) and colored according to their corresponding geographic area (in (d)). (c) A phylogenetic neighbor‐joining tree with tips colored by geographic area and enclosing circles representing generalized clades. (d) Geographical distribution of accessions with pie chart size representing the number of accessions collected in a particular country and slice size representing the probability of belonging to a specific genetic cluster. Dotted ellipses indicate the extent of the applied geographic areas (drawing in small groups of outliers; see Section 2 and Table S1 for further information on geographic area assignments). (e) A table of accessions assigned to geographic areas and genetic clusters; accessions without known location are designated as “Other.”

The analysis of genome‐wide genetic structure in finger millet revealed clear differentiation patterns (Figure 2). Both PCA and DAPC analyses (DAPC using an optimum PC number of 4 according to Figure S2 and an optimal *k* number of 4 according to Figure S3) revealed a genetic structure that corresponded with the four sampled geographic areas of finger millet cultivation (Figure 2a,b,d). Accessions from Africa and Asia regions were separated along the first PC, which explained 18.8% of the total variation, while the second PC, which explained 11.2% of the total variation, discriminated between geographic areas within the two continents (Figure 2a). The overlap between genetic clusters and geographic areas was particularly strong in Africa (Figure 2e; Table S4). The clear overall geographic structuring of genetic variation, with a degree of admixture, was also evident in the NJ tree (Figure 2c), on the map of sampled geographic areas showing accessions’ assignments to genetic cluster groups (Figure 2d), and in a heatmap of GRM with UPGMA clustering (Figure S6). The more extensive admixture of finger millet in Asia than Africa, most clearly observed in Figure 2d, is consistent with more formal breeding of the crop in Asia, supported by cross‐regional germplasm transfer. For example, in India, an improvement program initiated in the 1960s involved crosses between African and Asian accessions, resulting in “Indaf” varieties (Mirza & Marla, 2019; see more in next section). In further NJ analysis that considered SNPs for sub‐genomes A and B separately, clear geographic structuring of genetic variation was evident in both cases (Figure S7).

### 3.4 Genetic introgression and gene flow analysis suggests a migration event between East Africa and India

**FIGURE 3:**
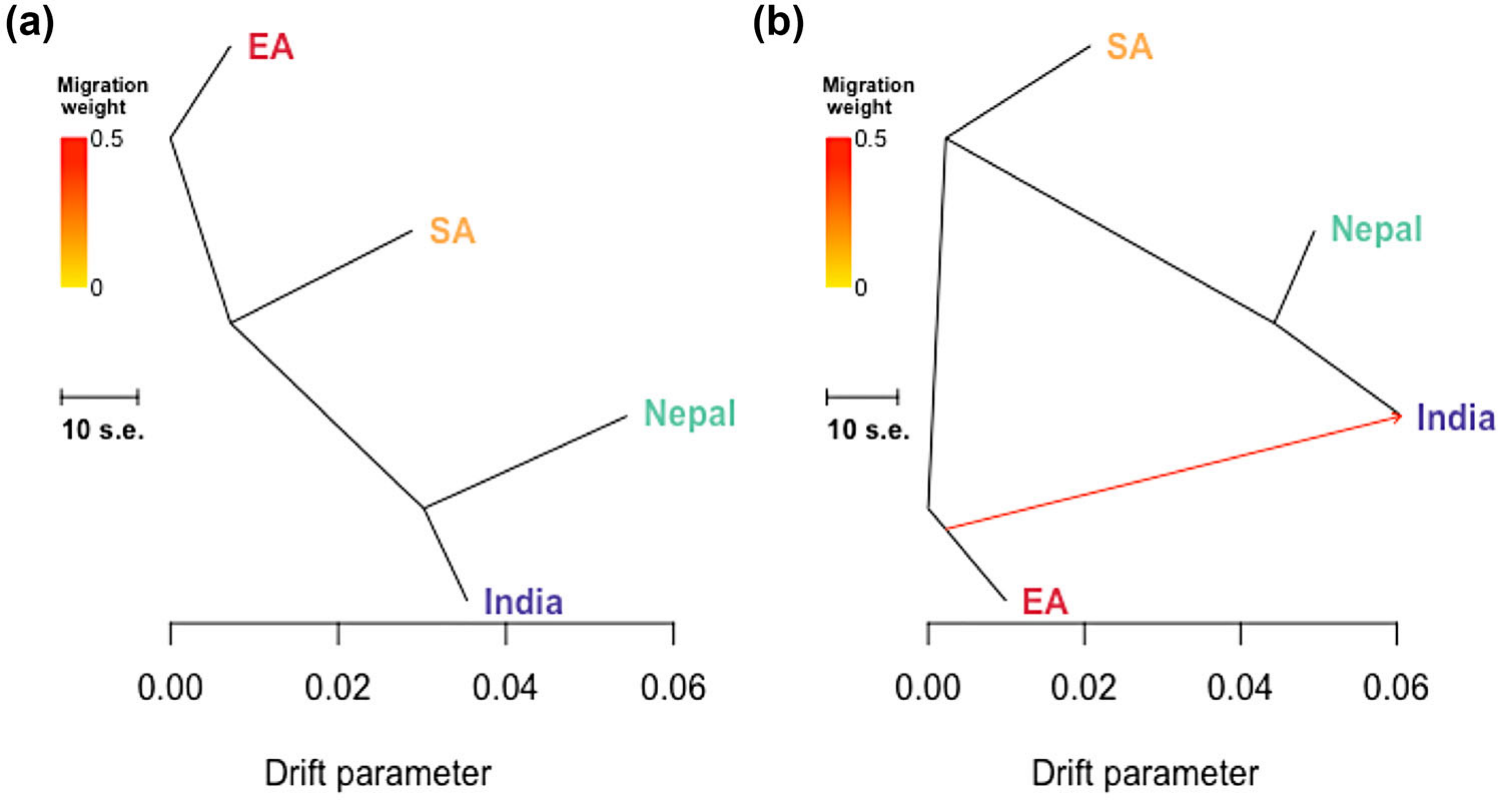
Inferred finger millet maximum‐likelihood population trees with admixture events. The structure of the graphs inferred by Treemix for four geographic areas of finger millet: (a) with no migration events; (b) with one migration event. The migration arrow is colored according to its weight and represents the fraction of ancestry derived from the migration edge. Horizontal branch lengths are proportional to the amount of genetic drift that has occurred on the branch. The scale bar shows 10 times the average standard error (s.e.) of the accessions in the sample. The residual fit from the graph is shown in Figure S8.

The Treemix analysis of past admixture events in finger millet suggested a potential historic introgression from East Africa to India. Using “East Africa” as an out‐group, the maximum‐likelihood tree without migration events (Figure 3a and Figure S8a), accounting for drift alone, corresponded to the phylogenetic tree (see Figure 2c). When migration events were allowed, a single event from “East Africa” to “India” was inferred (Figure 3b and Figure S8b). These results are consistent with the cultivated finger millet's demographic history as previously speculated by nongenomic analysis approaches (de Wet et al., 1984; Hilu & de Wet, 1976). These findings are also consistent with known breeding introductions from Africa to Asia (see previous section).

### 3.5 Chromosome‐level nucleotide diversity and differentiation profiles vary by region

The analysis of nucleotide diversity (π) along chromosomes showed differences in relative diversity for geographic areas by chromosome, including for homeologous (A and B sub‐genome) chromosomes (Figure S9a). Notable was relatively high diversity along chromosome 1A for “Nepal” (not seen on chromosome 1B) and along chromosomes 5B and 9B for “Southern Africa” (not seen on chromosomes 5A and 9A). Pairwise chromosome‐level *F*_ST_ values for geographic areas (Figure S9b) reflected these different diversity profiles, again indicating chromosome‐specific, sub‐genome‐based differences. Of note was high differentiation between both of the African geographic areas and both the Asian geographic areas along chromosome 5A that was not replicated on chromosome 5B.

### 3.6 Summarized gene diversity statistics confirm regional differentiation and sub‐genomic differences

The calculation of genome‐wide gene diversity (H) values indicated that the “Nepal” region contained the most diversity and the “East Africa” region the least (Table 2). The ranking of diversity levels corresponded when the analysis was repeated based on genetic clusters that approximate geographic areas. Estimates were, as expected, overall lower when based on genetic clusters, as the most prominent within‐area genetic admixture has in this case been reassigned (see Figure 2).

**TABLE 2:** Characterization of gene diversity (H). Genome‐wide measurement of finger millet gene diversity was undertaken for four geographic areas (left value) and corresponding genetic clusters identified by discriminant analysis of principal components (DAPC) analysis (right value). The values presented for individual sub‐genomes are for geographic areas only. The total sample size for genome‐wide estimates varies because of the nonassignment of some accessions to geographic areas (see Section 2). The area or cluster with highest diversity is underlined.

		H		
Geographic area/Genetic cluster	Sample size	Genome‐wide	Sub‐genome A	Sub‐genome B
India/Cluster 1	129/102	0.259/0.215	0.293	0.212
Nepal/Cluster 2	57/60	0.282/0.265	0.336	0.252
EA/Cluster 3	112/143	0.199/0.179	0.200	0.208
SA/Cluster 4	106/118	0.260/0.252	0.230	0.368

Pairwise *F*_ST_ values summarized for genome‐wide SNPs (Table S5) confirmed the PCA and DAPC results (Figure 2a,b,d), which revealed primary geography‐based partitioning between Africa and Asia and then between areas within these regions. The highest differentiation was between “Southern Africa” and “Nepal” accessions (*F*_ST_ = 0.167). The two AMOVA analyses revealed that 16% of total genome‐wide variation partitioned among the four geographic areas and 24% among the corresponding but genetically defined clusters (*p* ≤ 0.001 that no structuring in both cases).

In the case of gene diversity (H) calculations, geographic areas were also analyzed separately for A and B sub‐genomes (Table 2). The ranking of diversity by geographic area varied for the two sub‐genomes, with “Nepal” (still, compared to the entire genome) ranking highest for the A sub‐genome but “Southern Africa” highest for the B sub‐genome; “East Africa” consistently ranked lowest. The observed diversity ranking change is reflected in the relative spread of accessions from each geographic area in sub‐genome NJ trees (Figure S7). It appears to be based on changes in relative diversity for specific pairs of homeologous chromosomes (Figure S9a), notably chromosomes 5B versus 5A, and 9B versus 9A (diversity relatively high at 5B and 9B for “Southern Africa”; see also above).

### 3.7 Significant variation in phenotypic traits partitions by region

The analysis of BLUEs of 13 traits for 423 finger millet accessions revealed extensive variation (CV from 8.58 for threshing percentage to 48.25 for productive tiller number) and medium‐to‐high $H^{2}$ values (from 0.35 for grain yield to 0.95 for days to flowering). The level of variation detected and heritability values were generally consistent with the crop's previous field trials (e.g., Bharathi, 2011; Manyasa et al., 2016).

**FIGURE 4:**
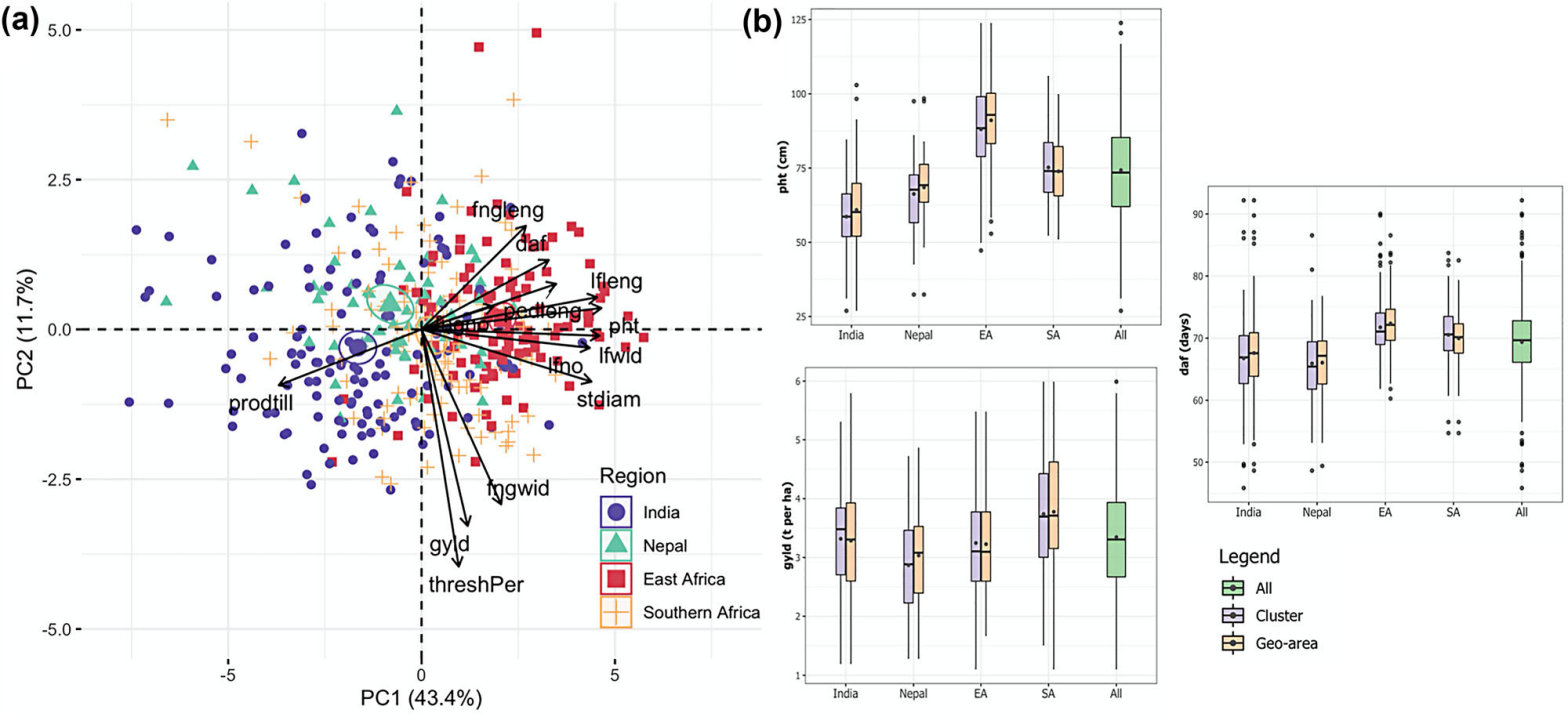
Summary of phenotypic data for 423 finger millet accessions. (a) The principal component analysis (PCA) biplot shows vectors of each of the 13 phenotypic traits/variables (black arrows) and the PCA scores for combined phenotypes of 404 accessions (represented by points) colored according to geographic areas; scores of 19 accessions without known location were excluded. The magnitude of the vectors shows the strength of their contribution to each PC. Vectors pointing in similar directions indicate positively correlated traits, vectors pointing in opposite directions indicate negatively correlated variables, and vectors at approximately right angles indicate low or no correlation. Colored ellipses represent 95% confidence intervals around the centroid (bold symbol) for each area. stdiam, stem diameter; lfleng, leaf length; lfwid, leaf width; lfno, leaf number; fngleng, finger length; fngwid, finger width; fngno, finger number; pedleng, peduncle length; pht, plant height; prodtill, production tillers; daf, days to flowering; thershPer, threshing percentage; gyld, grain yield. (b) Boxplots of the statistical distribution of individual trait values divided according to genetic clusters identified by discriminant analysis of principal components (DAPC) (purple boxplots), geographic areas (orange boxplots), and the total phenotypic variation (green boxplots). The middle bar and the point inside each boxplot represent median and mean, respectively. Examples are given for days to plant height (pht) and grain yield (gyld). Results for the other 10 phenotypic traits are shown in Figure S11.

Summary statistics are presented in Table S6, and trait distributions and between‐trait correlations in Figure S10. The strongest positive correlation was between leaf length and plant height (*r =* 0.83, *p* < 0.001), and the strongest negative correlation between leaf width and the number of productive tillers (*r* = −0.64, *p* < 0.001). Considering the three key traits of grain yield, plant height and days to flowering, a medium‐level positive correlation was observed between plant height and days to flowering (*r* = 0.50, *p* < 0.001), a moderate positive correlation between plant height and grain yield (*r* = 0.19, *p* < 0.001), and no correlation between days to flowering and grain yield (*r* < 0.01, NS). PCA using BLUEs of traits further illustrated the levels of correlation between different traits (Figure 4a).

Similar to genomic data, PCA scores of 404 accessions were used to examine the phenotypic data structure. Confidence ellipses based on geographic areas (Figure 4a; confidence level set to 95% around area centroid) indicated a degree of separation along the first PC for all four geographic areas based on combined phenotypes, most clearly separating “India” and “East Africa.” Accessions from “Nepal” and “Southern Africa” were relatively less differentiated compared to when using genome‐wide SNP data (see, e.g., *F*_ST_ values, Table S5). Trait‐specific boxplots of phenotypic variation by region or genetic cluster (Figure 4b and Figure S11) illustrated a divergent pattern for some traits. For example, for plant height “India” and “East Africa” (or their corresponding clusters) showed the most difference, while for grain yield “Nepal” and “Southern Africa” did. On the other hand, for days to flowering, the greatest difference was between “Nepal” and “East Africa.”

Of the three last‐mentioned traits, only plant height showed non‐overlap between areas/clusters (for “East Africa” vs. some other areas/clusters, where “East Africa” accessions were, e.g., on average 32% taller than accessions from “India,” Figure 4b; the same non‐overlap applied for the traits of leaf length, leaf number, leaf width [all greatest for “East Africa”] and number of productive tillers [fewest for “East Africa”], Figure S11). In general, phenotypes were more differentiated between regions (Africa vs. Asia) than among geographic areas within regions, corresponding to genomic differentiation (Figure 2) and consistent with combined phenotype centroids in Figure 4a. Overall, each geographic area contained extensive phenotypic variation, with India containing the most (Table S7; see also the PCA and the boxplots, Figure 4 and Figure S11).

### 3.8 Genome‐wide association analysis reveals a small number of significant associations and a range of candidate genes

GWAS detected 16 MTAs above the stringent Bonferroni threshold (‐log_10_(0.05/8778) = 5.24), 15 of which were chromosome‐located. Twelve were associated with finger length (seven on chromosome 2B, three on 5B, one on 7B and one on 8B), two with days to flowering (one on 4B and one scaffold marker), and two with threshing percentage (one each on 4B and 6B). Manhattan and Q‐Q plots for all 13 phenotypic traits are presented in Figure S12, and a list of all significant MTAs is given in Table S8. Most of the SNPs with significant MTAs that were identified by GWAS had a low MAF, in correspondence with the overall SNP panel (MAF < 0.2 for > 75% of all SNPs; see Figure S1d). Therefore, care should be taken when interpreting the findings. The relatively small number of MTAs that were detected could reflect the overall limited number of SNPs generated with the current genotyping strategy. All of the significant MTAs were associated with the B sub‐genome, even though it had a lower SNP density than the A sub‐genome (but the B sub‐genome does have slower LD decay, see above). A list of 61 unique putative candidate genes revealed within a 25 kb interval both upstream and downstream of significant MTAs is given in Table S8, but we do not here explore these associations further.

### 3.9 SNP effects clustering demonstrates different genetic architectures of phenotypic traits

**FIGURE 5:**
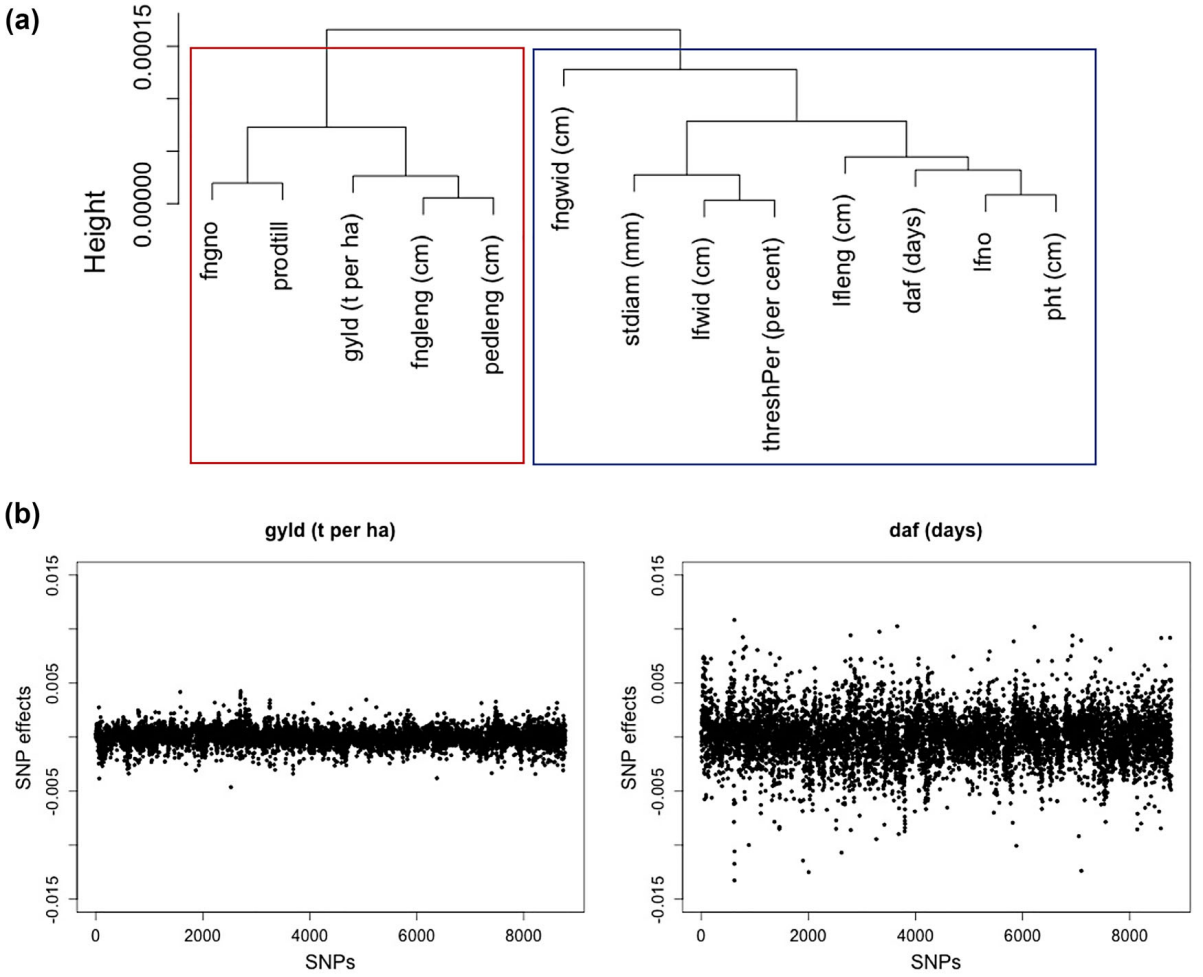
Clustering of single nucleotide polymorphism (SNP) effects obtained from ridge regression‐best linear unbiased genomic prediction (RR‐BLUP) to determine the complexity of agronomic traits. (a) The UPGMA clustering algorithm was applied to the five statistical moments of SNP effects for all 13 agronomic traits. The cluster in red consists of traits with highly polygenic genetic architecture, and the cluster in blue consists of traits with simple to moderately complex genetic architecture. (b) Examples of SNP effect distributions for the complex trait of grain yield (gyld; at left) and the less complex trait days to flowering (daf; at right). The results for the remaining 11 of the individual traits evaluated are shown in Figure S13.

The dendrogram ordering of SNP effects for phenotypic traits from RR‐BLUP revealed two clusters of traits of different levels of complexity (Figure 5 and Figure S13). Among traits with simpler genetic architecture were days to flowering and threshing percentage (consistent with MTAs that were detected above). These are traits that do not follow a uniform distribution. Included among the traits with more complex genetic architecture (and of more uniform distribution) were finger length (which did still reveal MTAs in GWAS analysis) and grain yield (no MTAs in the analysis and known in other cereals to be highly polygenic; e.g., for wheat, see Brinton & Uauy, 2019).

### 3.10 Comparison of phenotypic and genome‐wide gene diversity by geographic area supports varied post‐domestication diversification pathways for finger millet

**FIGURE 6:**
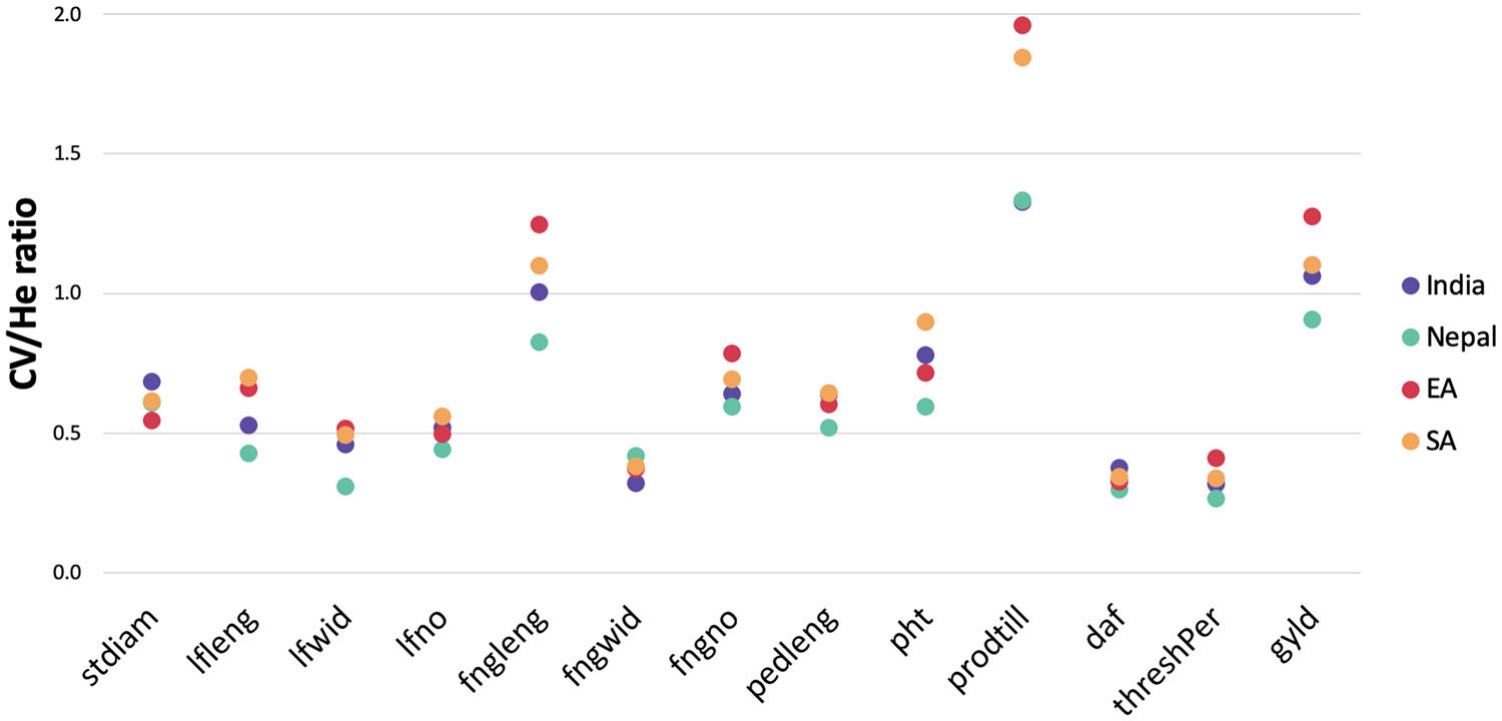
The ratio of the coefficient of phenotypic variance (CV) and genome‐wide gene diversity (H) computed for 13 agronomic traits across four geographic areas. The ratios were used to inform on finger millet's selection history.

A geographic‐area‐based comparison of phenotypic trait diversity with overall underlying gene diversity was based on the calculation of CV/H ratio (i.e., standardized phenotypic variation per unit of genome‐wide gene diversity; Figure 6). The results showed that the ranking of values between geographic areas varied by trait, suggesting complex, context‐specific diversification of the crop. The comparison of rankings of CV/H for finger number and plant height, for example, showed that the highest rank for the former was for “East Africa” and for the latter was “Southern Africa.” Considering all 13 phenotypic traits, “East Africa” most often of any geographic area ranked top for CV/H (in 6 cases) and “Nepal” bottom (11 cases). This is consistent with the relatively low denominator (H) value for “East Africa” compared to “Nepal” (see above), and possibly indicates an “overexpression” of phenotypic variation in the former region that is consistent with a longer domestication history. Days to flowering was the trait with the least spread in CV/H values for geographic areas, indicating that variation in this trait may be the best phenotypic proxy of underlying genomic diversity within geographic areas.

## 4 DISCUSSION

Broadening crop production requires more focus on orphan crops. Orphan crops have an intrinsic value, but they can also provide broader lessons on crop domestication and crop diversification pathways (Dawson et al., 2019). Here, we have studied the orphan crop finger millet and have shown that a combination of modern and traditional methods can provide important insights relevant for the future development of the crop.

Past DNA‐based studies of finger millet genetic diversity have generally been limited in scope, involving < 150 accessions and/or < 100 polymorphic loci. These studies include those of Dida et al. (2008), Arya et al. (2013), Manyasa et al. (2015), Ramakrishnan et al. (2016), Babu et al. (2018), Lule et al. (2018), and Pandian et al. (2018) who all applied simple sequence repeat (SSR) markers; and those of Gimode et al. (2016) and Sood et al. (2016) who used SSRs and SNPs. Despite their limited scope, these previous studies revealed localized and regional genetic variation, including differentiation between Africa and Asia, as well as instances of admixture between the two continents. A recent more extensive study by Devos et al. (2023) has also confirmed these findings.

A small number of previous studies have also sought to explore marker‐trait associations in finger millet. However, these studies also suffered from limitations in terms of the number of accessions and markers used and lacked a finger millet genome assembly to map markers to chromosome positions. For example, Dida et al. (2021) investigated blast disease resistance with a medium‐sized panel of SNPs but only included 52 East African accessions. Puranik et al. (2020) examined grain nutrient content‐related associations using a large panel of SNPs but assessed only 190 genotypes. Sharma et al. (2018) focused on 14 agro‐morphological traits with a medium‐sized panel of SNPs and only 113 accessions, while Tiwari et al. (2020) explored marker associations with grain protein content using the same SNPs and germplasm panel as Sharma et al. (2018). Recently, Sood et al. (2023) investigated major agronomic traits and blast resistance in 182 genotypes also using a medium‐sized panel of SNPs. Despite their limited scope, these previous studies identified certain marker‐trait associations, although the stringency and certainty levels varied, and some studies relied on candidate gene sequence homologies for characterization.

The analysis in our current study encompasses an extensive germplasm panel and a broad genome‐wide (and sub‐genome‐located) SNP marker set. This dataset was combined with phenotypic assessment in the field, the use of a finger millet genome assembly, and the application of various population genomic tools. Collectively, this analysis provides us with a more complete understanding of genetic variation in finger millet than has been previously achieved. Our analysis reaffirms the presence of geographically structured genetic variation, as observed in earlier studies. However, it also sheds further light on the evolutionary history of finger millet, revealing diverse pathways of diversification within and between regions. These findings can offer valuable insights to guide future breeding efforts and are in accordance with the other recent work of Devos et al. (2023), who used the same genome assembly and genotype‐by‐sequencing to assess the genomic characteristics of a panel of 294 *E. coracana* and three *E. indica* accessions.

Below, we focus further discussion on two aspects of particular interest that expand the current understanding of finger millet: (i) polyploidy and sub‐genome‐specific diversification; and (ii) geographically specific relationships between phenotype and genotype, embracing a range of individual phenotypic traits and combinations of traits, and chromosome‐, sub‐genome‐, and genome‐level diversity.

### 4.1 Sub‐genome‐specific diversification and polyploidy

Hybridization between different genomes, followed by chromosome doubling to generate polyploids, has played a crucial role in cereal crop evolution (Levy & Feldman, 2002). This has been well studied for major cereals such as allohexaploid wheat (Feldman & Levy, 2012). However, the full origin of the finger millet allotetraploid genome is not well understood (Zhang et al., 2019). The allopolyploidization event from diploid A and B sub‐genome progenitors to form the tetraploid crop ancestor (*E. corocana* subsp. *africana*) is believed to have occurred in East Africa (de Wet et al., 1984). Traditionally, such polyploidization events are thought to be rare in plant's histories and initially lead to genomic bottlenecks in the derived organisms (Stebbins, 1950). In the current study, relative diversity levels of the cultivated finger millet could not be compared with the descendants of its wild tetraploid progenitor or previous diploid progenitors. However, previous studies found genomic diversity in cultivated finger millet to be significantly lower than in wild *E. coracana* subsp. *africana* (e.g., Gimode et al., 2016). We were nevertheless able to search for sub‐genomic and chromosome‐specific diversity patterns in finger millet by geographic area of sampling, which may inform on its evolution. The highest level of genetic diversity was detected in the B sub‐genome overall in “Southern Africa,” with exceptionally high diversity levels along two chromosomes (5B and 9B). In contrast, for the A sub‐genome, “Southern Africa” only ranked third in diversity, while diversity for “East Africa” ranked lowest for both sub‐genomes.

These observations are consistent with the broadening of diversity in specific parts of the finger millet genome as the crop developed new adaptations during expansion from East Africa to new areas in Africa and in Asia. This may have been facilitated by the polyploidization process that enables mechanisms such as inter‐genomic transfer through translocation, recombination, and transposition to support rapid evolution (as outlined by Levy & Feldman, 2002). Our results could also indicate a secondary contact in the “Southern Africa” region between the crop and its immediate progenitor, or perhaps new introgressions from other co‐located *Eleusine* species that have specifically targeted the B sub‐genome and could have been aided by polyploidization. Both wild *E. coracana* subsp. *africana* (i.e., the considered most likely immediate progenitor) and a range of other *Eleusine* species are sympatric with the finger millet crop in southern Africa (Figure 1), providing opportunities for introgression (see also discussion in Devos et al., 2023). This type of process has been observed in other cereals, including wheat (He et al., 2019), and would be consistent with genotyping‐by‐sequencing in finger millet that has revealed chromosomal rearrangements between sub‐genomes (Qi et al., 2018). Regardless of the cause, the patterns of sub‐genome‐specific diversity observed suggest complexity in finger millet's evolution. Further genomic exploration of *Eleusine* germplasm panels, containing extant descendants of known and putative crop wild progenitors, may further elucidate this complex demographic history.

### 4.2 Region‐specific genomic–phenotypic relationships

Our analysis shows complex relationships between genomic and phenotypic variation in finger millet across the four geographic areas sampled. This is consistent with a crop that has been subject to millennia of domestication and has experienced different selection pressures based on particular human preferences and production environments in different locations. Complex relationships between genetic variation and phenotypic variation are common in crops (e.g., Kozak et al., 2011), and these results were therefore not unexpected.

The analysis of genetic diversity along sub‐genomic chromosome homeologs indicated differences in relative diversity for particular geographic areas. The subsequent pooling of chromosome diversity at the sub‐genome level also revealed different diversity rankings by location. When levels of geographic‐area‐based phenotypic variation were compared with genome‐wide gene diversity (through the calculation of CV/H), the results suggested context‐, trait‐, and trait‐combination‐specific diversification pathways for the crop. In addition, the “East Africa” area most often ranked top for CV/H values, consistent with a longer cultivation history here than elsewhere. The profiles of phenotypic traits alone that varied between geographic areas by individual trait further suggest multiple selection pressures both between and within areas, with the broad variation often captured within areas indicating local differential selection.

Finally, patterns of differentiation between geographic areas also varied for genomic data compared to phenotypic data, further supporting complex demographic processes. For example, genome‐wide SNPs revealed the highest differentiation between “Southern Africa” and “Nepal” accessions, but combined phenotypic data indicated the greatest level of difference between “India” and “East Africa.” This perhaps illustrates that these last two areas are the strongest foci for context‐specific crop development and/or are most agro‐ecologically differentiated.

### 4.3 Concluding remarks

This study has revealed distinct genomic and phenotypic variation patterns in finger millet that suggest complex diversification pathways for the crop. These findings provide a robust foundation for the future breeding‐based advancement of finger millet. One practical application is the potential identification of heterotic groups, which can guide the production of hybrid finger millet varieties. By leveraging hybridization between “East Africa” and “Nepal” or “Southern Africa” accessions, valuable genetic variation can be introduced into the genetically narrower East Africa region. The substantial phenotypic and genetic variability observed overall suggests that significant initial genetic improvements can be achieved in finger millet breeding, even without relying on molecular markers. To gain a more comprehensive understanding of the crop's demographic history, it is necessary to conduct further studies, involving broader *Eleusine* germplasm panels that encompass known and putative crop wild progenitors. Additionally, future GWA studies may need to consider larger populations with more extensive phenotypic data from multiple locations and higher SNP coverage across the genome, including rare variants, to overcome the limitations in discovering MTAs as was the case in this study. The identification of precise causal genomic regions may remain challenging in finger millet due to the relatively slow decay of LD (although see Devos et al., 2023). Last, as genomic resources continue to develop for finger millet, breeding material selection approaches that assign genomic estimated breeding values to each separate sub‐genome may also open a possibility for weighted sub‐genome selection.

## AUTHOR CONTRIBUTIONS

**Jon Bančič**: Conceptualization; data curation; formal analysis; investigation; methodology; validation; visualization; writing—original draft; writing—review and editing. **Damaris A. Odeny**: Conceptualization; data curation; methodology; project administration; resources; writing—review and editing. **Henry F. Ojulong**: Resources; writing—review and editing. **Samuel M. Josiah**: Resources. **Jaap Buntjer**: Methodology. **R. Chris Gaynor**: Methodology. **Stephen P. Hoad**: Supervision; writing—review and editing. **Gregor Gorjanc**: Supervision; writing—review and editing. **Ian K. Dawson**: Conceptualization; investigation; methodology; supervision; writing—original draft; writing—review and editing.

## CONFLICT OF INTEREST

The authors declare no conflict of interest.

## Supporting information

Supplemental Tables Information

Supplemental Figures Information

## Data Availability

All data generated and analyzed during this study are included in this published article and its supplemental material. The raw phenotypic and DArTseq SNP data were deposited into Dryad Digital Repository (http://datadryad.org/).
